# Atypical central retinal artery occlusion as the first presentation of POEMS syndrome: a case report

**DOI:** 10.1186/s12883-018-1071-y

**Published:** 2018-05-08

**Authors:** Panitha Jindahra, Charungthai Dejthevaporn, Pimjai Niparuck, Jariya Waisayarat, Piyaphon Cheecharoen, Thanatporn Threetong, Purit Petpiroon, Tharikarn Sujirakul, Anuchit Poonyathalang, Kavin Vanikieti

**Affiliations:** 1 Department of Medicine, Faculty of Medicine Ramathibodi Hospital, Mahidol University, 270 Rama VI Road, Bangkok, 10400 Thailand; 2 Department of Pathology, Faculty of Medicine Ramathibodi Hospital, Mahidol University, 270 Rama VI Road, Bangkok, 10400 Thailand; 3 Department of Radiology, Faculty of Medicine Ramathibodi Hospital, Mahidol University, 270 Rama VI Road, Bangkok, 10400 Thailand; 4 Department of Ophthalmology, Faculty of Medicine Ramathibodi Hospital, Mahidol University, 270 Rama VI Road, Bangkok, 10400 Thailand

**Keywords:** POEMS syndrome, Central retinal artery occlusion, Anterior ischemic optic neuropathy, Visual loss

## Abstract

**Background:**

POEMS syndrome is a plasma cell disorder, which clinically manifests from paraneoplastic syndrome: polyneuropathy, organomegaly, endocrinopathy, monoclonal plasma cell disorder, and skin changes. The most common ocular manifestation is optic disc swelling, whereas other ocular manifestations; cystoid macular edema, serous macular detachment, venous sinus thrombosis, infiltrative orbitopathy, uveitis, neovascularization of the disc, peripapillary choroidal neovascularization and optic disc drusen, had also been reported.

**Case presentation:**

A 52-year-old Thai man presented with 5-day sudden painless visual loss in the left eye. Ocular examination revealed visual acuity of 20/20 and no light perception in the right and left eye, respectively. Right fundoscopic examination was significant for hyperemic generalized optic disc swelling. Left fundoscopic examination revealed opaque and edematous entire retina giving the appearance of central retinal artery occlusion (CRAO) along with pallid “chalky white” optic disc swelling. Fluorescein angiography showed profound leakage of bilateral optic nerve heads and arteriolar filling defect in macular area along with leakage of small retinal arterioles in the left eye. Indocyanine green angiography demonstrated choroidal filling defect in the left eye only. Neuroimaging showed enhancement and luminal narrowing of left internal carotid artery, early subacute watershed infarctions in the left cerebral hemisphere and pachymeningeal enhancement. Cerebrospinal fluid analysis revealed high protein level with normal opening pressure. Intravenous methylprednisolone was initially started without any benefit. After extensive investigations, diagnosis of “POEMS syndrome” was made based on polyneuropathy, elevated lambda light chain level, elevated plasma vascular endothelial growth factor (VEGF), hepatomegaly, spinal sclerotic bone lesions, and thrombocytosis. Furthermore, sural nerve biopsy demonstrated neuropathy and positive VEGF staining. He was treated with eight cycles of bortezomib, cyclophosphamide and dexamethasone (BorCyDex). Polyneuropathy and thrombocytosis had remarkably improved after 2nd cycle, whereas, visual impairment had shown no recovery. Hepatomegaly was significantly reduced after the completion of BorCyDex. Our case eventually received autologous hematopoietic stem cell transplantation with high dose melphalan.

**Conclusions:**

To our knowledge, we illustrated the first patient given CRAO as the first presentation and ocular finding ever reported in POEMS syndrome. Both cerebral and ocular infarctions were presumably the result of VEGF-induced cranial vasculopathy as evidenced by neuroimaging.

## Background

POEMS syndrome is a plasma cell disorder, which clinically manifests from paraneoplastic syndrome: polyneuropathy, organomegaly, endocrinopathy, monoclonal plasma cell disorder, and skin changes [1]. The most common ocular manifestation is optic disc swelling, whereas other ocular manifestations; cystoid macular edema (CME), serous macular detachment, venous sinus thrombosis, infiltrative orbitopathy, uveitis, neovascularization of the disc (NVD), peripapillary choroidal neovascularization and optic disc drusen, had also been reported [2–12]. To our knowledge, central retinal artery occlusion has never been reported in POEMS syndrome. We herein describe the first reported patient with POEMS syndrome who initially presented with atypical central retinal artery occlusion.

## Case presentations

A 52-year-old Thai man presented with sudden painless visual loss in his left eye upon awakening 5 days prior to presentation. He had no visual complaints regarding his right eye. He reported no fever, jaw claudication, general malaise, anorexia, hearing of a whooshing sound, or new-onset headache. His ocular history was significant for bilateral hyperopia. His medical history was notable for 18.5-kg body weight loss in the past 2 years. He was not taking any medications and was a nonsmoker.

**Fig. 1:**
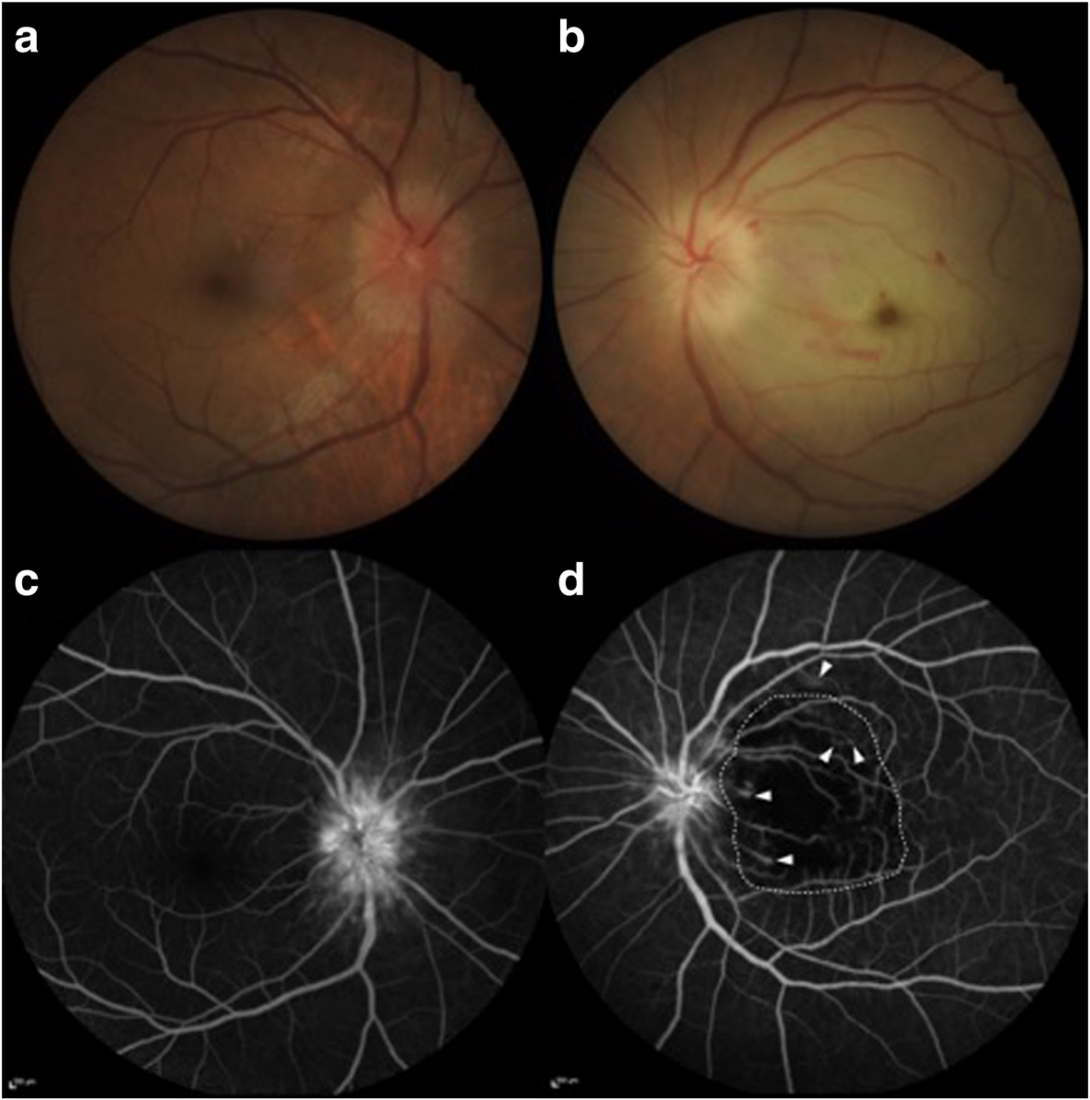
Fundus photograph and fundus fluorescein angiography. **a** Hyperemic and generalized optic disc swelling is observed in the right eye. **b** An opaque and edematous retina in the posterior pole along with pallid “chalky white” generalized optic disc swelling is demonstrated in the left eye. **c** Fundus fluorescein angiography showing profound leakage of the optic nerve head in the right eye. **d** Leakage of the optic nerve head, filling defects in multiple arterioles predominantly in the macular area (*area within the dashed lines*), and leakage of small retinal arterioles (*arrowheads*) are observed in the left eye

Ocular examination revealed visual acuity of 20/20 and no light perception in the right eye and left eye, respectively. The presence of a grade 4+ relative afferent pupillary defect of the left eye was noted. The intraocular pressure was 15 mmHg bilaterally. Slit-lamp examination showed a mild cortical cataract without signs of ocular inflammation bilaterally. Fundoscopic examination of the left eye showed that the entire retina was opaque and edematous, particularly in the posterior pole, and pallid “chalky white” generalized optic disc swelling was noted (Fig. 1b). Neither emboli nor cotton-wool spots were detected. Fundoscopic examination of the right eye was significant only for hyperemic generalized optic disc swelling; no hemorrhage, cotton-wool spots, or exudates were present (Fig. 1a).

A color vision test (Lanthony desaturated 15-hue test) of the right eye was unremarkable. The Humphrey 24-2 automated static perimetry program of the right eye showed blind spot enlargement. The average retinal nerve fiber layer thickness of the optic nerve head measured by spectral-domain optical coherence tomography (Cirrus HD-OCT, software version 6.0.0.599; Carl Zeiss Meditec, Inc., Dublin, CA, USA) was 554 and 187 μm in the right and left eye, respectively. Fundus fluorescein angiography (FFA) revealed profound leakage of the bilateral optic nerve heads, and filling defects were observed in multiple arterioles, predominantly in the macular area of the left eye, along with leakage of small retinal arterioles in the left eye (Fig. 1c, d). No filling defect or retinal vessel leakage was noted in the right eye. Indocyanine green angiography (ICG) demonstrated a choroidal filling defect in the left eye only. Partial occlusion of both retinal and choroidal circulation in the left eye was suspected. An electroretinogram (ERG) was evaluated according to the International Society for Clinical Electrophysiology of Vision standard (MonColor; Metrovision, Perenchies, France). A full-field ERG of the left eye showed a significant reduction of both the a-wave and b-wave amplitude, along with a decreased b:a ratio with a normal implicit time in the photopic and maximal combined responses, confirming both retinal and choroidal circulation in the left eye was affected. An ERG of the right eye was normal. Both sides of the superficial temporal artery were pulsatile and painless. The patient’s blood pressure was 130/80 mmHg. A provisional diagnosis of atypical central retinal artery occlusion (CRAO) with anterior ischemic optic neuropathy (AION) in the left eye in conjunction with generalized right optic disc swelling was made. Treatment was started with intravenous methylprednisolone at 1 g once daily for 5 days.

A complete blood count showed 5.7 × 10^9^/L white blood cells with 76% neutrophils; the platelet count was 595× 10^9^/L, and the hemoglobin level was 17.3 g/dL. The erythrocyte sedimentation rate was 9 mm/h, and the C-reactive protein level was 1.14 mg/L. The results of a comprehensive vasculitis panel, including measurement of antinuclear antibody, cytoplasmic antineutrophil cytoplasmic antibodies, perinuclear antineutrophil cytoplasmic antibodies, and anti-double-stranded DNA, were within normal limits. The results of the serum Venereal Disease Research Laboratory (VDRL) test, *Treponema pallidum* hemagglutination (TPHA) test, and anti-human immunodeficiency virus antibody test were negative. A metabolic panel, including liver function tests, the fasting blood sugar level, and a lipid profile, was unremarkable. Antiphospholipid antibodies were negative. The results of a coagulation panel were within normal limits.

**Fig. 2:**
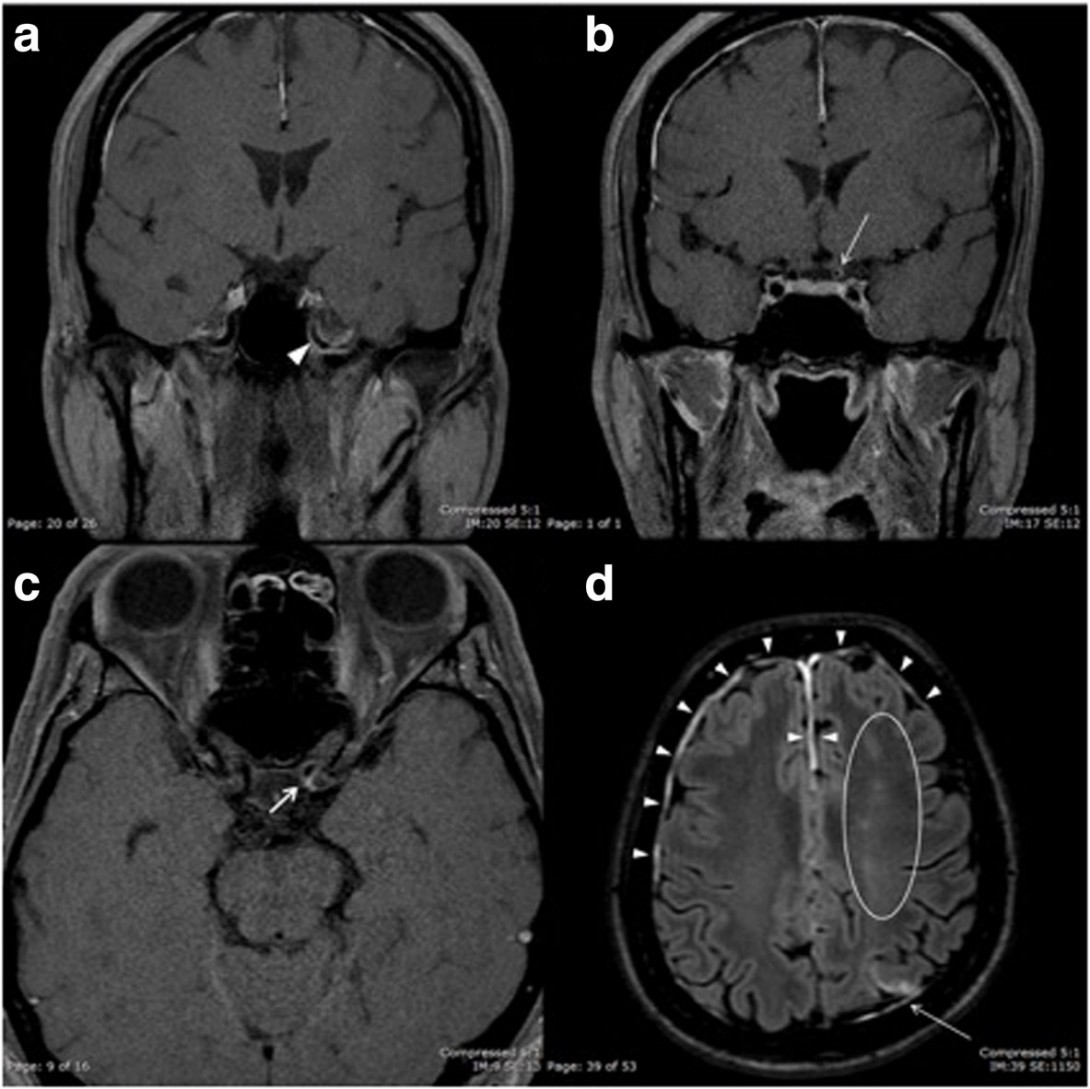
Magnetic resonance imaging of the brain with gadolinium injection. **a**, **b** Coronal T1-weighted images with gadolinium injection showing enhancement and luminal narrowing of the petrous (*arrowhead*) and supraclinoid (*arrow*) left internal carotid artery. **c** Axial T1-weighted image with gadolinium injection showing enhancement and luminal narrowing of the supraclinoid (*arrow*) left internal carotid artery. **d** Axial fluid attenuation inversion recovery (FLAIR) image with gadolinium injection showing early subacute left anterior cerebral artery/middle cerebral artery watershed infarction (*area within the circle*), early subacute left middle cerebral artery/posterior cerebral artery watershed infarction (*arrow*), and marked pachymeningeal thickening with enhancement of the bilateral cerebral convexities (*arrowheads*)

Magnetic resonance imaging (MRI) of the brain with gadolinium injection revealed early subacute left anterior cerebral artery/middle cerebral artery watershed infarction, early subacute left middle cerebral artery/posterior cerebral artery watershed infarction, and marked pachymeningeal thickening with enhancement of the bilateral cerebral convexities (Fig. 2d). Diffusion weighted imaging (DWI) of the brain showed restricted diffusion involving gray-white matter of the left parietal lobe and centrum semiovale. Moreover, enhancement and luminal narrowing of the petrous and supraclinoid left internal carotid artery (ICA) were observed (Fig. 2a–c). The bilateral optic nerves showed a normal size and signal intensity. Magnetic resonance angiography (MRA) revealed luminal narrowing from the distal cervical left ICA extending to the supraclinoid left ICA. The patient was treated with aspirin. Echocardiography and electrocardiography showed normal results.

A lumbar puncture was then performed, and 6 mL of clear cerebrospinal fluid (CSF) was obtained with an opening pressure of 19 cmH_2_O. CSF analysis revealed a significantly high protein level (234.6 mg/dL), normal glucose concentration (61 mg/dL), and white cell count of 4 cells/mm^3^ (100% mononuclear cells). The VDRL test, TPHA test, polymerase chain reaction (PCR), and cultures for microorganisms were all negative in the CSF.

No clinical improvement was demonstrated during the 5-day course of methylprednisolone, and patient developed numbness in both feet.

**Fig. 3:**
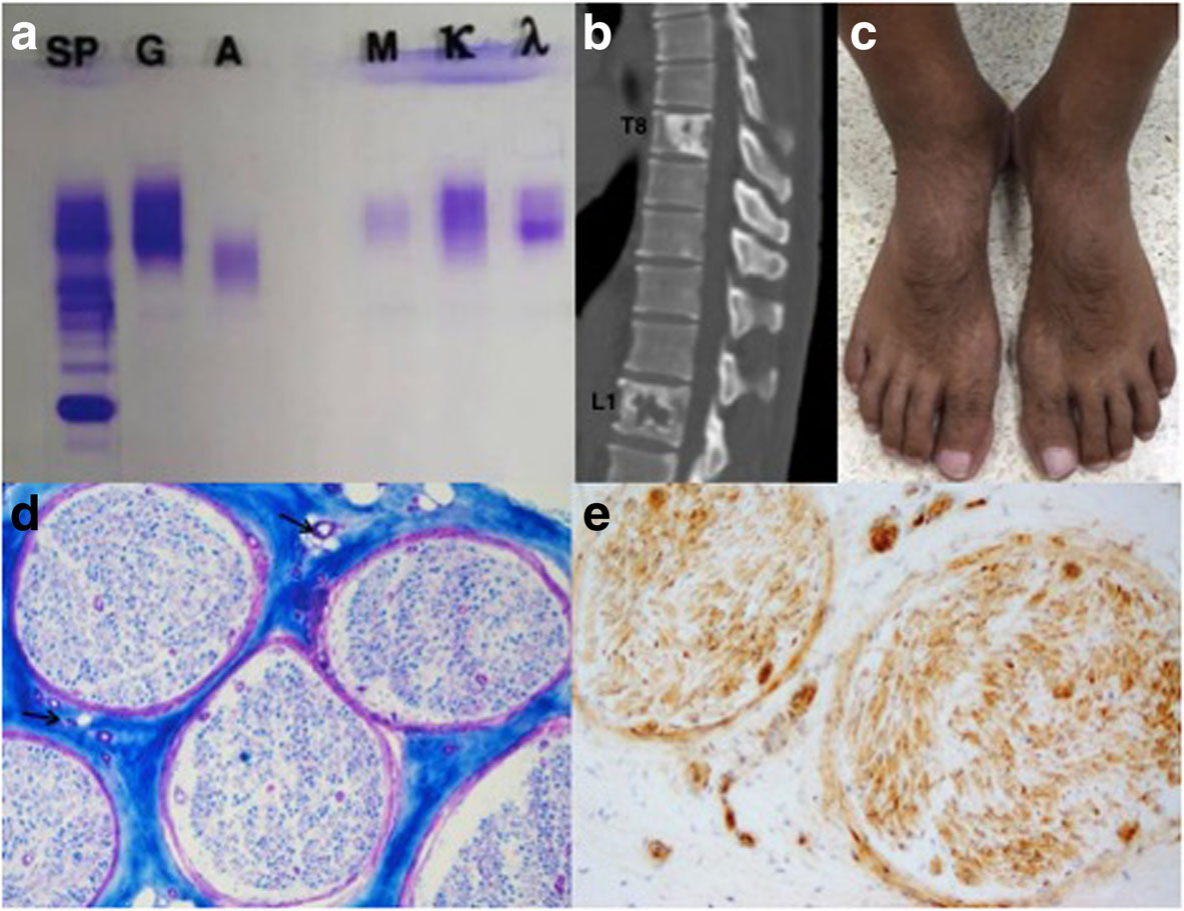
Serum immunofixation electrophoresis, computed tomography of the spine, external appearance of both feet, and sural nerve biopsy. **a** Serum immunofixation electrophoresis showing an IgGλ monoclonal gammopathy. **b** Computed tomography of the spine with contrast showing multiple mixed lytic osteosclerotic bone lesions involving the T8 and L1 bodies. **c** Localized hypertrichosis associated with hyperpigmentation involving the dorsal aspect of both feet. Sural nerve biopsy showing neuropathy through slightly decreased myelinated fibers density with mild to moderate increase of small vessels in epineurium (arrows) (**d**: Luxol Fast Blue staining, 100×) and positive VEGF staining (**e**: VEGF staining, 200×)

Serum protein electrophoresis revealed no hypergammaglobulinemia, whereas, an IgGλ monoclonal gammopathy was detected by serum immunofixation electrophoresis (Fig. 3a). In addition, computed tomography (CT) of the whole spine showed multiple mixed lytic osteosclerotic bone lesions involving the T8 and L1 bodies (Fig. 3b), bilateral 8th ribs, right ilium, and right sacrum.

A more comprehensive systemic examination showed a symmetrical decrease in the motor power of the bilateral extensor hallucis longus and a symmetrical decrease in sensation (including proprioception, vibration, pain, and temperature) of both feet. The Achilles and patellar reflexes were absent bilaterally. A nerve conduction study also revealed sensory and motor demyelinating polyneuropathy. The Romberg sign was positive and steppage gait was noted. Moreover, hepatomegaly was observed on a CT scan of the whole abdomen, and localized hypertrichosis associated with hyperpigmentation involving the dorsal aspect of both feet was noted (Fig. 3c).

A diagnosis of POEMS syndrome was made. The levels of hormones, plasma interleukin-6, and plasma vascular endothelial growth factor (VEGF) were measured and PCR for human herpesvirus type 8 in serum was performed to support this diagnosis. The interleukin-6 level was < 1.5 pg/mL (reference range, 0–7 pg/mL), and the plasma VEGF level was 1828 pg/mL (reference range, 0–860 pg/mL). PCR for human herpesvirus type 8 in serum was negative. The levels of hormones, including testosterone, free T3, free T4, thyroid-stimulating hormone, and cortisol, were normal.

A bone marrow biopsy demonstrated normocellular bone marrow; adequate red cells, white cells, and platelets; and scattered plasma cells. CD138 immunohistochemistry showed only 5% plasma cells.

Sural nerve biopsy was also undertaken and demonstrated neuropathy through slightly decreased myelinated fibers density (Fig. 3d). Mild to moderately increased small vessels in epineurium led to the diagnosis of POEMS syndrome, which confirmed by a positive VEGF (Fig. 3e). There was no evidence of vasculitis or amyloidosis. The patient was treated with eight cycles of bortezomib, cyclophosphamide, and dexamethasone (BorCyDex). After the 2nd cycle of BorCyDex, the polyneuropathy had remarkably improved and the platelet count returned to normal; however, the visual loss in the left eye was still present. Disease assessment was performed after the 4th and 8th cycles of BorCyDex. CSF was obtained and showed high protein levels (200 and 150 mg/dL after the 4th and 8th cycle of treatment, respectively). Hepatomegaly was significantly reduced based on CT. After eight cycles of BorCyDex, the patient finally underwent autologous hematopoietic stem cell transplantation with high-dose melphalan.

## Discussion and conclusions

Our patient was diagnosed with POEMS syndrome based on demyelinating polyneuropathy, lambda light chain-restricted monoclonal plasma cell proliferative disorder, sclerotic bone lesions, plasma VEGF elevation, hepatomegaly, hypertrichosis associated with hyperpigmentation, thrombocytosis, and bilateral optic disc swelling [1].

To our knowledge, this is the first reported patient with POEMS syndrome to exhibit CRAO as the first presentation. Optic disc swelling is the most common ocular finding and occurs in 52.0 to 67.5% of affected patients [2, 3].

Other ocular manifestations reported in the literature include cystoid macular edema [4–6], serous macular detachment [7, 8], venous sinus thrombosis [9], infiltrative orbitopathy [10], uveitis [11], neovascularization of the disc [10], peripapillary choroidal neovascularization, and optic disc drusen [12].

Our patient was considered to have atypical CRAO given the demonstration of small retinal arteriole leakage on FFA, association with AION, and contralateral generalized optic disc swelling.

Our hypothesis regarding the mechanism causing leakage of small retinal arterioles on FFA in the present case is microvascular hyperpermeability induced by an increased plasma VEGF level. VEGF is thought to be the major contributor to various manifestations of POEMS syndrome, including ocular findings [1, 2, 6, 13].

The association with AION was the second atypical characteristic of CRAO in this case. The typical pallid “chalky white” generalized optic disc swelling led us to highly suspect paraoptic short posterior ciliary artery involvement. Inability to perceive light was caused by acute ischemia of not only the retina but also the optic nerve, which is supplied by the choroidal circulation. This was confirmed by a full-field ERG, which showed a significant reduction in both the a-wave and b-wave amplitude. Moreover, indocyanine green angiography of the left eye revealed a choroidal filling defect, consistent with short posterior ciliary artery occlusion. Ophthalmic artery occlusion was the most likely explanation for the ischemia of both the retina and optic nerve. This was supported by the findings of luminal narrowing of the left ICA on MRI and MRA.

The last atypical characteristic of CRAO was the generalized optic disc swelling in the right eye. Given the well-preserved visual function of the right eye despite the appearance of marked swelling of the optic disc, arteritic AION of the right eye was less of a concern. Considering the normal CSF opening pressure, the cause of the optic disc swelling in the right eye was probably microvascular hyperpermeability induced by the increased plasma VEGF level. This speculation is consistent with many previous reports [1, 2, 6, 13]. However, the pathophysiology of optic disc swelling remains unclear.

Cerebral infarctions in patients with POEMS syndrome have been previously described [14–18]. The area of infarction varies and may include the end arteries, watershed areas, and cervical and proximal intracranial vasculature [14]. Potential etiologies remain inconclusive. Dupont et al. [14] reported that an elevated platelet count and evidence of plasma cell proliferation on bone marrow biopsy lead to an increased risk of ischemic stroke. These findings are consistent with the present case.

In our patient, vasculopathy of the left ICA based on MRI and MRA could explain both the cerebral infarctions and the CRAO associated with AION. Moreover, hyperviscosity secondary to thrombocytosis was thought to be the synergic risk factor that led to the arterial thrombosis events.

Arterial walls enhancement has rarely been reported. Fu et al. [18] described a patient with POEMS syndrome who developed multiple acute watershed infarctions of the left cerebral hemisphere associated with enhancement and luminal narrowing of the ipsilateral ICA. These findings were similar to those in our patient. Moreover, generalized cranial pachymeningeal involvement was observed in our patient. This is consistent with a previous study reported by Briani et al. [19], who described nine patients with POEMS syndrome who showed pachymeningeal thickening with intense enhancement. Furthermore, two of nine patients showed hyperplasia of meningothelial cells, neovascularization, noninflammatory obstructive vessel remodeling, and strong coexpression of VEGF and VEGF receptor on meningeal biopsy. These findings suggest that VEGF might be a major contributor in the pathogenesis of pachymeningeal involvement in POEMS syndrome. We speculate that the possible cause of arterial wall enhancement might be similar to that of VEGF-induced pachymeningeal enhancement. However, we did not perform a biopsy of any affected vasculature or meninges.

With respect to the vasculopathy of the left ICA based on MRI and MRA and leakage of the bilateral optic nerve heads and small retinal arterioles in the left eye on FFA, our patient demonstrated involvement of all sizes of affected arterial vessels ranging from the distal cervical ICA to small retinal arterioles. Elevation of the plasma VEGF level is proposed to be the major contributor to these abnormalities.

Sural nerve biopsy is recommended at baseline if clinically indicated [1]. Symmetrical decrease in sensation of both feet was exhibited in our patient. This was supported by a nerve conduction study, which revealed sensory demyelinating polyneuropathy. Similarly, a reduction in the myelinated fiber population was revealed pathologically. In addition, VEGF overexpression and VEGF-related proliferation of endothelial cells as evidenced by the biopsy supported the diagnosis of POEMS syndrome [1].

In conclusion, we have herein reported the first patient with CRAO as the initial presentation and ocular finding of POEMS syndrome. The mechanism of occlusion remains unclear due to its rarity. Further studies are needed to determine the definite pathophysiology.

## Abbreviations

AION: Anterior ischemic optic neuropathy
BorCyDex: Bortezomib, cyclophosphamide, and dexamethasone
CME: Cystoid macular edema
CRAO: Central retinal artery occlusion
CSF: Cerebrospinal fluid
CT: Computed tomography
ERG: Electroretinogram
FFA: Fundus fluorescein angiography
ICA: Internal carotid artery
ICG: Indocyanine green angiography
MRA: Magnetic resonance angiography
MRI: Magnetic resonance imaging
NVD: Neovascularization of the disc
PCR: Polymerase chain reaction
TPHA: *Treponema pallidum* hemagglutination
VDRL: Venereal disease research laboratory
VEGF: Vascular endothelial growth factor
